# Sea urchin waste as valuable alternative source of calcium in laying hens’ diet

**DOI:** 10.1371/journal.pone.0314981

**Published:** 2025-03-04

**Authors:** Francesca Leone, Michela Sugni, Stefania Marzorati, Silvia Rizzato, Lorenzo Ferrari, Paolo Tremolada, Valentina Ferrante

**Affiliations:** 1 Department of Environmental Science and Policy, Università degli Studi di Milano, Milan, Italy; 2 Department of Chemistry, Università degli Studi di Milano, Milan, Italy; Tokat Gaziosmanpaşa University: Tokat Gaziosmanpasa Universitesi, TÜRKIYE

## Abstract

Annually, 3000–3500 tons of sea urchins are harvested in the Mediterranean Sea, with only their gonads being consumed (10–30% of the total weight), leaving the rest as waste. This waste, consisting of the skeleton, is rich in biominerals, mainly calcium with a small amount of magnesium, and contains potent antioxidant compounds. Considering the issues of resource overconsumption, and in line with the circular economy concept, this study explored the potential of replacing limestone-derived calcium with sea urchin waste in the diet of laying hens, which require this element to produce eggs. The experiment involved two groups of hens: one receiving a control diet containing limestone-derived calcium, and another fed an experimental diet containing sea urchin waste as alternative source of calcium. Parameters such as egg production, animal welfare, egg quality, and bone breaking strength were assessed. Additionally, the antioxidant activity and carotenoid content of the diets were evaluated, revealing no significant differences between the two groups. Both groups exhibited similar egg production rates; however, the treated group had a lower percentage of discarded eggs and fewer lesions on the head, back, and tail, indicating better animal welfare. The treated group produced eggs with significantly thicker shells, though no significant differences were observed in eggshell weight, breaking strength, ultrastructure. There were also no differences in yolk colour or antioxidant activity between the two groups. Similarly, no significant differences were found in tibia breaking strength, confirming that sea urchin waste can provide bioavailable calcium without compromising bone quality. In conclusion, substituting limestone-derived calcium with sea urchin waste in laying hens’ diets positively affected animal welfare and improved eggshell thickness without compromising egg quality, suggesting that sea urchin waste can be recycled as a valuable alternative to limestone-derived calcium in laying hens’ feed. However, further research is necessary to confirm these findings.

## Introduction

Humanity is consuming the planet’s resources at a rate 50% faster than their regeneration capacity, leading to critical reflection on resource utilization [1]. Currently, a possible solution is circular economy, defined as the sharing, reuse, repair, renovation, and recycling of existing materials and products, including food waste [2]. Among the latter, sea food by-products can account to relevant amounts, mainly as a result of processing and packaging from industries.

In the Mediterranean Sea, 3000–3500 tons of the edible sea urchin *Paracentrotus lividus* are harvested annually for food consumption [3–5]. Italy is the main consumer among European countries, with 30 million individuals annually collected from the wild [6]. However only the gonads, accounting approximately to 10–30% of the total weight of the animal, are consumed, while the remaining pars, mainly composed of the skeleton (test +  spines), is discharged as waste [7]. As highlighted by Garau et al. [8], this disposal is neither environmentally nor economically sustainable.

Considering that sea urchin tests and spines contain large amounts of biominerals (the *skeleton* is composed by calcium carbonate in the form of calcite, with a high percentage of magnesium carbonate [9] they can be incorporated into sustainable feed formulations for laying hens, which actually require a high amount of calcium (Ca) to produce eggs. In fact, Ca is one of the most important minerals in laying hens’ diet, playing an essential role in metabolism, bone development, and eggshell formation [10]. The eggshell consists of 94–97% calcium carbonate (CaCO_3_), arranged in calcite crystals [11]. Magnesium (Mg), present in eggshells in the form of magnesium carbonate (MgCO_3_), is the mineral with the second highest concentration. Its supplementation has been shown to increase eggshell strength and thickness, improving eggshell quality [12]. Normally, the major source of Ca in animal feed is limestone. However, one of the major problem with this source is the percentage of Ca it contains, as this is relatively low and rather variable: indeed, it can vary from 32 to 38% [13], thus containing a high amount of residues which can affect its digestibility. When the absorbed calcium is insufficient, the percentage of discarded eggs increases. Damaged or broken eggs account for 10–15% of all eggs laid, causing economic losses for farmers [14]. An inadequate amount of Ca leads not only to smaller eggs and poorer eggshell quality, but also to skeletal abnormalities, reducing bone mineral content and increasing bone fragility [15]. Also a magnesium deficit causes thin eggshells and an apparent reduction in plasma calcium levels associated with decreased bone mobilization [16,17]. Nutritional insufficiency of these two elements affects animal welfare.

In recent years, minerals of biological origin (biominerals) have been shown to be more bioavailable and usable in lower quantities [14]. They are more easily absorbed and retained by birds, which also reduces the excretion of minerals that pollute the environment [18]. Some researchers have tested oyster shell [19,20], but the results reported in literature are inconsistent. Moreover, oyster shells can contain some potentially toxic elements such as aluminium, cadmium, and mercury [20].

Additionally, besides Calcium, sea urchin tests and spines contain potent antioxidant compounds (both a specific class of small polyphenols called polyhydroxynaphthoquinones and carotenoids from gonad residues) [7], therefore its inclusion in hens’ diet may provide bioactive molecules potentially improving the diet quality and hens’ productivity (e.g., egg quality). Considering the growing importance of applying a circular economy approach, this study aims to verify if sea urchin waste can be efficiently re-used as a source of calcium (and bioactives) in laying hens’ diet. Specifically, the objective was to evaluate the potential of introducing sea urchin waste-derived calcium (S-Ca) into the diet of laying hens as a substitute for limestone-derived calcium (L-Ca), without negatively impacting the welfare of the hens, egg quality, and eggshell ultrastructure. In addition to calcium, this study also aimed to assess whether sea urchin-derived waste could serve as a source of magnesium and other biologically active compounds in the diet of laying hens.

## Materials and methods

### Sea urchin waste collection and processing

A total of approximately 400 kg of frozen sea urchin waste was collected from sea urchin processing SMEs in Sardinia and Sicily, as well as from restaurants in Apulia, Italy (see S1 Table and S2 Table). This waste was subsequently transferred to a specialized company for drying (30 minutes at 80 °C) and grinding (final size comparable to limestone granules). A small aliquot was used for evaluation of nutritional values, element analysis and Ca/Mg solubility (S3 File), this information being fundamental for the subsequent diet production. The final grinded product was then included in the experimental diet as described below.

### Animals and housing

The study involved no procedures causing animal suffering was conducted in the facilities of the University of Milan (Lodi, Italy). The facilities are compliant with the Directive 1999/74/EC, which sets minimum standards for the protection of laying hens. The research involved 128 Hy-Line Brown hens, housed in 16 enriched cages from 19^th^ to 52^nd^ week of age. At the end of the production cycle, the hens were sent to a slaughterhouse and, after passing veterinary inspection, were intended for human consumption and donated to charity. The animals were controlled every day for health, behaviour and welfare.

The experimental groups were as follows:

- **control group**: 64 animals, divided into 8 cages (8 hens per cage), which received a commercial diet with limestone-derived calcium;
- **treated group**: 64 animals, divided into 8 cages (8 hens per cage), fed on an experimental diet supplemented with sea urchin waste.

A photoperiod of 16L:8D was applied. The temperature was maintained at around ± 20°C, the relative humidity at about 55%, and the light intensity at around 100 lux. Feed and water were provided ad libitum. This study was carried out in strict accordance with the recommendations in the Guide for the Care and Use of Laboratory Animals of the National Institutes of Health. The protocol was approved by the Organismo Preposto al Benessere degli Animali (OPBA) of the University of Milan (Protocol Number: 31_2021, released May 7^th^, 2021).

### Diet composition and characterization

The nutritional composition of two diets is reported in Table 1, and their analytical components in Table 2. The experimental diet was specifically produced taking into account the analytical and mineral components, as well as Ca and Mg solubility of sea urchin waste (S3 File), so that the final nutritional value and calcium amount could be similar between the two diets. Furthermore, both diets were characterized in terms of carotenoid content and antioxidant activity (see below and S4 File and S5 File).

**Table 1:** Composition of the two diets.

Components	Control Diet (%)	Treated Diet (%)
Corn meal	42.37	41.78
Soft wheat	20	20
Dehulled soybean meal 46%	20.31	19
Sea urchin waste	/	10.8
Sunflower seed meal 30%	3.5	3.5
Soybean oil	2	2.5
Calcium phosphate	1.2	1.2
Rovimix ovoxan	0.5	0.5
Sodium chloride	0.22	0.22
L-lysine	0.2	0.2
DL-methionine	0.2	0.2
Sodium bicarbonate	0.1	0.10
Calcium carbonate	9.4	/

**Table 2:** Analytical composition of the two diets.

Components	Control Diet (%)	Treated Diet (%)
Crude protein	16.33	15.81
Crude fats	4.56	5.02
Crude fiber	3.06	3.00
Crude ash	12.92	13.72
Calcium	3.94	3.87

#### Carotenoids quantification in diets.

To quantify the carotenoids present in both types of feed (control and treated) and compare them, a modified procedure from the literature was adopted [21]. A calibration curve was first constructed using a sample of a standard carotenoid (lutein, Merck, 90% purity). A stock solution of lutein in acetone was prepared at a known concentration of 0.916 mg/mL. From this, successive dilutions in acetone were prepared. The absorbance of each solution was measured at 447 nm using a spectrophotometer (LANGE DR-3900) and plotted against the concentration of standard lutein (mg/mL). Both feed samples (control and treated) were finely pulverized using mortars. A precise mass of 1.060 g from each sample was weighed and placed inside two 50 mL plastic tubes. To each tube, 10 mL of an ethanol (1:1) solution was added to solubilize the carotenoids. The solutions were kept under magnetic stirring for 24 hours at room temperature. The next day, an aliquot of the supernatant solution was taken after centrifugation (6000 rpm for 5 minutes). The absorbance of each supernatant solution was measured at 447 nm and using the equation of the calibration line of standard lutein, the total carotenoids present in each feed were calculated as lutein equivalents (mg_LUTEIN_/g_FEED_). Results are reported in Supporting Information (S4 File).

#### Antioxidant activity of diets.

To evaluate the antioxidant capacity of each diet, the radical cation 2,2’-azino-bis (3-ethylbenzothiazoline-6-sulfonic acid) (ABTS^•+^) was employed, following the procedure used by Loganayaki et al. [22] and Marzorati et al. [7], with slight modifications. Initially, an aqueous solution containing 36.6 mg of ABTS^•+^, and 6.5 mg of ammonium persulfate (Merck) was prepared in a 10 mL flask and left at room temperature in the dark for one day. The ABTS^•+^ solution was then diluted in ethanol to achieve an absorbance of approximately 0.7 AU at 734 nm (approximately 1:75 v/v).

Subsequently, cuvettes containing 1.2 mL of the diluted ABTS^•+^ solution were mixed with increasing concentrations of the previously prepared supernatant solutions extracted from the two feed samples. These cuvettes were kept in the dark for 1 hour, after which the absorbance was measured at 734 nm using a spectrophotometer. A blank cuvette devoid of any sample was used as reference. The percentage of remaining ABTS^•+^ was calculated as: % remaining ABTS^•+^ =  (A_734 nm, 1 h, sample_/A_734 nm, 1 h, blank_) ×  100%, and the results were graphically plotted against the concentration of the feed extract in the solution. Results are reported in Supporting Information (S5 Fig).

### Data collection

#### Animal welfare.

Animal welfare was assessed at the beginning, middle, and at the end of the trial (19^th^, 38^th^, and 51^st^ week of age). The European Welfare Quality^®^ Protocol [23], which evaluates the condition of feathers, keel bone, and footpads of the hens, was used. The assessment consisted in assigning a score ranging from 0 to 2 to the head, back, tail, cloaca, and comb, based on the severity of lesions:

- score 0: no damage;
- score 1: a damaged area of less than 5 cm or fewer than 3 lesions;
- score 2: a damaged area greater than 5 cm or more than 3 lesions.

For footpads, scores also ranged from 0 to 2:

- score 0: no lesions;
- score 1: presence of necrosis or epithelial proliferation or bumblefoot without dorsal swelling;
- score 2: dorsal swelling.

The keel bone was examined visually and by running fingers alongside and over it to the end of the bone. Scores assigned were:

- score 0: linear;
- score 1: slightly to moderately prominent keel, not sharp, with flat breast muscle;
- score 2: severely prominent keel, with depressed contour to breast muscle.

Additionally, each hen was weighed at each timepoint

#### Bone breaking strength.

At the end of the cycle, 3 right tibia per cage were sampled at the slaughterhouse to measure their breaking strength, serving as an indicator of adequate mineral supplementation and animal welfare [16]. The bones were previously frozen and then conditioned overnight at 20 °C. A TA.HDplus Texture Analyser (Stable Micro Systems Ltd., UK), equipped with a 500 N load-cell and controlled by the specific software Texture Exponent TEE32 (v. 3.0.4.0, Stable Micro Systems Ltd., UK), was utilized for the analysis.

The traverse speed was set at 20 mm/min, and a wsxc deformation was applied to the center of the bones, using a support span distance of 60 mm. The results are expressed as the mean and standard deviation of breaking load (N). Additionally, considering variations in bone dimensions, the results were standardized based on minimum diameter, maximum diameter, and support span distance [24].

#### Performance.

Egg production and percentage of discarded eggs were recorded daily.

The egg production was calculated with the following formula:


$$\frac{\text{number of eggs laid weekly per group}}{\text{number of hens present x }7}\times 100$$

The percentage of discarded eggs was calculated with the following formula:


$$\frac{\text{number of eggs discarded weekly per group}}{\text{number of eggs produced weekly per group}}\times 100$$

#### Egg quality.

***Weight*.**

During the trial, every month 3 eggs per cage were collected. The total weight of each egg and the weight of its components (eggshell, albumen, and yolk) were measured using a digital balance. The weight of the albumen (g) was calculated by subtracting the combined weight of the yolk and eggshell (g) from the total egg weight (g).

***Yolk colour.***On the same eggs, the yolk colour was measured using a DSM-Firmenich digital colorimeter (Digital YolkFan™), which objectively assigns a score on the Roche scale. A lens reads the yolk colour positioned on a flat white surface and communicates the result to a mobile phone application.

***Antioxidant activity of the yolk.***

The determination of antioxidant activity in the yolks was conducted at the 39^th^ week using 16 eggs, and at the 51^st^ week using 8 eggs. According to Omri et al. [25], approximately 3 g of yolk mass was weighed for each sample and transferred quantitatively into 50 mL plastic tubes containing 10 mL of acetone. After manual shaking for about 1 minute, the tubes were centrifuged at 6000 rpm for 10 minutes. The supernatant was separated and tested for its antioxidant activity. The same procedure previously applied for determining the antioxidant activity of the feed was performed. EC_50_ values were determined and compared.

***Eggshell thickness.*** To assess the influence of calcium source on eggshell thickness, measurements were taken at three points on the acute pole using a digital caliber, and the mean of these three measurements was calculated.

**Eggshell breaking strength.** At the 39^th^ and 51^st^ weeks, the breaking strength of 10 eggs per cage was measured using an Instron dynamometer (model 3365, Instron Italia, Turin, Italy) operated with “Bluehill Universal” software. A 100 N load cell was mounted on the instrument, connected to a probe with a plate diameter of 2.5 cm. The traverse speed was set to 20 mm/min, and deformation was applied to the equator of the egg, up to a maximum of 1 mm, positioning the egg along its major axis. Results were expressed as mean ±  standard deviation in terms of breaking load (N).

***Eggshell ultrastructure (SEM).***

The ultrastructure of the eggshell was analyzed using 8 samples (4 controls +  4 treated) collected at the 42^nd^ week and 6 samples (3 controls +  3 treated) collected at the 51^st^ week. These samples were taken from eggs previously subjected to mechanical testing to explore potential mechanical-structural correlations.

Analyses were conducted on the inner side of the eggshell at the acute pole (area of maximum curvature). Eggshell caps, approximately 1 cm² each, were rinsed in distilled water, boiled in 0.5 M NaOH for 10 minutes, thoroughly rinsed again with distilled water, and air-dried. Subsequently, they were mounted on stubs, gold-sputtered, and observed under a Scanning Electron Microscope (FE-SEM SIGMA Zeiss). Five random photos were taken for each eggshell sample.

***Purity and magnesium content of eggshell***.

Conventional powder X-ray diffraction technique (XRD) was used to characterize the crystalline structure and the composition of eggshells that are made essentially of calcium carbonate (about 95%), in the form of calcite, containing small amount of magnesium (until 3%). The breaking strength results were used to select the most different eggshells for this analysis. The eggshell material was washed by immersion in ethanol for 20 minutes and dried in oven at 40 °C overnight. The whole eggshell was then finely grounded in a mortar and a small portion was deposited on a flat sample holder of glass. Ambient X-ray powder diffraction patterns were collected on a series of samples using a Miniflex-600 diffractometer (Rigaku, Japan) with Cu K_α_ (λ =  1.540598 Å) radiation over the angular range of 10–60° (2θ), with an incremental step size of 0.02° (2θ) and a counting time of 1 s × step^-1^. Preliminary phase identification was performed by comparing the experimental data to that stored in reference databases (COD and PDF-2) [26,27]. The spectra were then refined and analysed to quantify the magnesium content, as described in the Supporting Information (S6 File).

### Statistical analyses

Welfare evaluation (feathers, footpad and keel bone) was examined with cross table analyses, considering the observed and expected cases for the two treatment and the three time-point. The statistical analysis used Likelihood Ratio when the expected frequencies were very low (<5) and the total case number were lower than 200.

The tibia breaking strength in treated and control group was analyzed with t-test.

Egg quality (weights of eggs and their components and eggshell thickness) was checked for normal distribution and tested with General Linear Model (GLM), which is based on multifactorial analysis of variance. Egg quality parameters were considered as dependent variables, while treatment and time were considered as factors. The interaction between factors was also evaluated by GLM, together with marginal means and 95% confidence interval.

Yolk colour was examined with cross table analyses, considering the observed and expected cases (colour score of Scala Roche) for the two treatments. The statistical analysis used Likelihood Ratio.

The eggshell breaking strength in treated and control group was analyzed with t-test.

All the analyses previously cited were performed with the statistical software SPSS 29.0.1.0.

Data collected from the content of carotenoid and the antioxidant activity of the diet and of the yolk were analyzed with Excel.

Purity and magnesium content of eggshell data were analyzed with Excel, applying ANOVA-one way.

All the effects were considered statistically significant at the threshold of p-value <  0.05.

## Results and discussion

### Animal welfare

Animal welfare was checked at the 19^th^, 38^th^, and 51^st^ week, however at the beginning of the trial no lesions were observed, so the 19^th^ week was excluded from the statistical analyses. The results are shown in Table 3, where are reported the observed frequencies in the 3 different scores.

**Table 3:** Results of welfare evaluations.

Week	Treatment	Head			P-value
		0	1	2	
38	C	62	0	0	0.221
	T	55	1	0	
51	C	41	11	6	0.031
	T	45	4	1	
Week	**Treatment**	**Back**			**P-value**
		0	1	2	
38	C	30	20	12	0.038
	T	39	13	4	
51	C	28	17	13	0.005
	T	39	7	4	
Week	**Treatment**	**Tail**			**P-value**
		0	1	2	
38	C	37	25	0	0.459
	T	34	21	1	
51	C	32	12	14	0.026
	T	35	12	3	
Week	**Treatment**	**Cloaca**			**P-value**
		0	1	2	
38	C	60	2	0	0.092
	T	52	1	3	
51	C	56	2	0	0.644
	T	49	1	0	
Week	**Treatment**	**Comb**			**P-value**
		0	1	2	
38	C	26	29	7	0.010
	T	37	18	1	
51	C	36	21	1	0.230
	T	37	11	2	
Week	**Treatment**	**Footpad**			**P-value**
		0	1	2	
38	C	38	24	0	0.022
	T	45	10	1	
51	C	27	30	1	0.927
	T	25	24	1	
Week	**Treatment**	**Keel bone**			**P-value**
		0	1	2	
38	C	44	16	2	0.646
	T	42	11	3	
51	C	32	24	2	0.253
	T	32	14	4	

Each hen per treatment was also weighed and then evaluated. The results of weigh-ins showed no significant differences between the treated and control group. As expected, the weight trend increased similarly during the cycle in both treated and control group: at T0, 1460 ± 133.9 g and 1504 ± 146.4 g (mean±SD; p =  0.083), at T1, 1695 ± 309.5 g and 1758 ± 224.3 g (mean±SD; p =  0.208), and at T2, 1868 ± 246.1 g and 1873 ± 232.2 g (mean±SD; p =  0.918), due to the aging of animals.

### Feather evaluation

The evaluation of feather condition involved different areas of the body where hens tend to peck, such as head, back, tail, and cloaca. Results obtained showed an increase in lesions on the head, back, and tail over time (Table 3). At the 51^st^ week, a significant difference in head evaluation was found between the two groups: hens receiving the experimental diet reported a lower frequencies of score 1 and 2 than the control group (Likelihood Ratio =  6.95; df =  2; p =  0.031). A similar result was observed in tail evaluation, where the frequencies of score 2 assigned at the 51^st^ week were significantly lower in treated hens (Likelihood Ratio =  7.26; df =  2; p =  0.026). Also, in back evaluation, the scores 1 and 2 were assigned more frequent to the control group, indicating a better condition of the back feathers of the treated group, both at 38^th^ week (Likelihood Ratio =  6.554; df =  2; p =  0.038) and 51^st^ week (Likelihood Ratio =  10.534; df =  2; p =  0.005). Regarding the cloaca (Table 3), no significant differences were found in the frequencies observed neither at 38^th^ week (Likelihood Ratio =  4.765; df =  2; p =  0.092) nor at the 51^st^ week (Likelihood Ratio =  0.214; df =  1; p =  0.644).

As regards comb evaluation, a significantly lower frequency of score 1 and 2 was found in hens fed the experimental feed at the 38^th^ week (Likelihood Ratio =  9.286; df =  2; p =  0.010), with no statistical differences at the end of the trial (Likelihood Ratio =  2.938; df =  2; p =  0.230).

Welfare Quality® [23] includes evaluation of feather condition, as feather pecking is a frequent issue in laying hen farms. This behavioural problem involves the removal of feathers from one bird by another, causing acute and chronic pain, reducing egg production, and increasing mortality [28–30]. It is influenced by factors such as light intensity, animal density, gut microbiota, inadequate space for foraging, insufficient material for scratching or dust bathing, and nutritional deficiencies or imbalances [31–34].

In this study, supplementation of sea urchin waste in laying hens’ diet appeared to improve feather condition, though the specific mechanism remains unknown. As mentioned before, numerous factors can contribute in the development of this behaviour [35].

### Footpad lesions

As part of the animal welfare evaluation, footpad lesions were also assessed. As the hens aged, the condition of their footpads deteriorated, due to the housing system, which in the present study were enriched cages. Hens fed the experimental diet showed lower frequency of footpad lesions compared to the control group (Table 3). However, only at the 38^th^ week there was a statistical (Likelihood Ratio =  7.612; df =  2; p =  0.022).

Footpad dermatitis (FPD), first reported in 1980, is a type of contact dermatitis characterized by inflammation and necrotic lesions on the plantar surface of the footpad. It ranges from superficial to deep ulcers and abscesses, often associated with bacterial infections and gangrenous dermatitis, causing acute and chronic pain. Footpad lesions are included in animal welfare assessments as indicators of housing conditions and overall poultry welfare [36].

Similar to feather pecking, several factors contribute to the development of FPD, including litter quality, litter depth and moisture, diet composition, drinker design, genetic predisposition, stocking density, weather conditions, and management practices [36]. Nevertheless, the results of footpad evaluations in this study suggest that replacing L-Ca with S-Ca does not adversely affect animal welfare.

### Keel bone deviation

Considering the crucial role of calcium in bone mineralization and structure, keel bone deviations were evaluated in the present study. No significant differences were found in the observed cases of the treated and control group (Table 3), neither at the 38^th^ week (Likelihood Ratio =  0.874 df =  2; p =  0.646) nor at the 51^st^ week (Likelihood Ratio = 2.749; df =  2; p =  0.253).

Keel bone deformities represent a welfare concern. These deviations from the normal straight shape can result from painful fractures, posing a welfare risk. Minor deviations may stem from decalcification or pressure from perches on the keel bone [23]. The ossification process of the keel bone continues for several weeks after initial deposition (approximately until 40 weeks of age), during which the caudal tip remains largely cartilaginous due to the substantial calcium demands for eggshell formation [37]. Therefore, sufficient intake of bioavailable calcium is crucial for both production and animal welfare. However, factors beyond calcium, such as genetics, age, housing systems, and perch design, can also influence keel bone deviations [38]. Hens in cage systems tend to have more fragile bones due to limited movement [39]. Conversely, in multi-tier aviaries, hens can move and perch on multiple levels, potentially increasing the risk of keel bone fractures [38].

In present study, the absence of significant differences between the treated and control group indicates a positive outcome, suggesting that the diet containing sea urchin waste did not adversely affect keel bone health.

### Bone breaking strength

To verify if sea urchin waste supplied bioavailable calcium without deteriorating bone structures, the breaking strength of the tibia was measured. The tibia was chosen because it contains a significant proportion of medullary tissue, where calcium is sequestered for eggshell formation [10]. In the present study, breaking strength was expressed as the mean breaking load±SD (150.3 ± 34.5 N and 156.2 ± 29.9 N for treated and control group, respectively): no significant differences were found between the two groups (t =  −0.64; df =  45; p =  0.530). These breaking strength values are higher than those reported in other studies on laying hens housed in enriched cages (120–140 N) [40,41].

The few studies in the literature that involve the replacement of L-Ca with biological sources (such as oyster shells) reported varying results: while Guinotte and Nys [42] and Saunders-Blades et al. [18] did not report any influence on tibia breaking strength, Fleming et al. [43] observed an improvement in this parameter using oyster shell as a calcium source. In the present study, the absence of differences in tibia breaking strength between the two groups is a successful outcome. This, combined with the keel bone evaluation, appears to confirm that sea urchin waste can provide bioavailable calcium without compromising bone quality and, consequently, animal welfare.

### Performance

#### Egg production.

**Fig 1:**
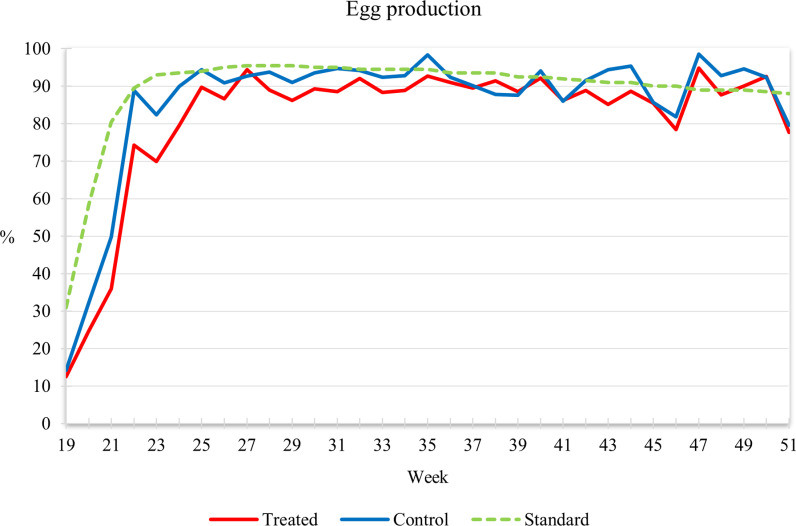
Egg production.

The deposition curve is presented in Fig 1, showing the percentage of deposition each week during the trial for both the treated and control group, compared to the Hy-Line Brown standard. Initially, production increased exponentially during the first period, but both groups were slightly below the hybrid standard. The peak was reached around the 24^th^ week, after which production stabilized until the 51^st^ week, when a physiological decrease occurred. The control group maintained higher production compared to the treated group, following the Hy-Line standard. In contrast to these findings, Kismiati et al. [44] reported an increase in egg production with the use of biologically derived calcium from eggshells at 7.5%. Similarly, Alm et al. [33] observed higher production in hens fed with 8% calcium from oyster shells, although not statistically significant. Saunders-Blades et al. [18] and Lee et al. [45] found no differences in egg production among hens fed different calcium sources, such as oyster shells. Additionally, Cufadar et al. [13], Frontng and Bergquist [46], Gongruttananun [47], and Islam and Nishibori [20] supported these findings, indicating no adverse effects on performance using biological sources of calcium.

#### Discarded eggs.

The treated group exhibited a lower percentage of discarded eggs compared to the control (2.10% vs 2.38%), suggesting a potential positive effect of S-Ca on this parameter. However, other factors can influence the percentage of discarded eggs, such as the age of the animals, their health status, and environmental conditions [48].

### Egg quality

#### Weight.

The egg weight is a critical parameter used to assess its quality [49] and determines its commercial classification. Typically, it ranges between 50–70 g and correlates with the weight of the hen; thus, as hens age, eggs tend to increase in both size and weight [50,51]. The results of the statistical analyses of egg and component weights are presented in Table 4. Data were analyzed by GLM considering treatment and time as factor together with their interaction. During the trial (time factor), the whole egg weight, albumen, and yolk weight slightly changed significantly (see Table 4), as expected. For this reason, the mean weights in treated and control group were reported as marginal mean with 95% confidence interval, which took into account time variation. Time and treatment interaction for the whole egg, albumen and yolk was not significant (F_7_;_368_ =  0.5; p =  0.835. F_7_;_368_ =  0.4; p =  0.902. F_7_;_368_ =  0.5; p =  0.856), confirming that the significant effect of the treatment was not affected by time. However, eggshell weights did not significantly differ between the two groups, even if there was a time effect (F_7_;_368_ =  8.4; p <  0.001) and time and treatment interaction (F_7_;_368_ =  3.2; p =  0.002).

**Table 4:** Marginal mean with 95% confidence interval of eggs and components weight (g).

Parameter	Marginal mean with 95% confidence interval (g)		F^a^	D. F.^a^	P-value^a^
	Control group	Treated group			
*Whole egg*	60.8 (60.3–61.4)	59.3 (58.7–59.8)	16.4	1; 368	<0.001
*Eggshell*	8.3 (8.2–8.4)	8.2 (8.1–8.4)	0.4	1; 368	0.554
*Albumen*	37.1 (36.7–37.6)	36 (35.5–36.5)	11.9	1; 368	<0.001
*Yolk*	15.4 (15.2–15.7)	15 (14.8–15.3)	5.0	1; 368	0.026

^a^Fisher’s F values, degree of freedom (D.F.), and associated probability (p-value) of the treatment factor, calculated by GLM.

As indicated in Table 4, the whole egg, albumen and yolk of the treated group were significantly lighter than those of the control group, which contrasts with the findings of Kismiati et al. [44], who reported significantly higher egg weights by adding calcium from eggshells at 7.5%. However, various factors influence egg weight, including breed, age, temperature, and high laying intensity, which negatively correlates with egg size [49–52]. Despite being statistically significant, the observed differences are slight and, most of all, both groups’ eggs belong to the same commercial category (size M).

#### Yolk colour.

Yolk colour is typically measured on the Roche scale, ranging from 1 to 15 [53]. In this study, the higher score frequencies were 9 in both treated and control group (respectively, 45% and 50%). The second colour score more frequent was 10 (respectively, 27% and 22%). Frequencies distribution in observed colour (from 7 to 12) were not significantly different in the two groups (Likelihood Ratio =  2.74; df =  5; p =  0.741). Yolk colouration is an important parameter because it influences consumers’ perception of egg quality worldwide. Consumers generally prefer yolks with intense colours [54], which depend on the accumulation of carotenoids obtained through diet or synthesized by the animals [53]. Since Marzorati et al. [7] identified carotenoids (echinenone, astaxanthin, and β-carotene) in sea urchin waste, this analysis aimed to determine whether the experimental feed affected final yolk colour compared to the control feed.

One possible explanation is that the antioxidants present in the sea urchin waste were degraded during the heat treatment required for drying (see Materials and methods paragraph), resulting in lower quantities than previously identified by Marzorati et al. [7]. Alternatively, these antioxidants may not be well absorbed in the intestine. The values obtained in both groups are lower than the consumer preference standard (12–14), indicating that no additional pigments were added to the diets.

#### Antioxidant activity of the yolks.

EC_50_ value was calculated for yolks of both treated and control samples, at two different timepoints, half and end of the experimental trials. The results expressed as value±SD are reported in Table 5.

**Table 5:** Results from ABTS^�+^ antioxidant activity.

	*39*^*th*^ *week*		*52*^*nd*^ *week*	
	Control group	Treated group	Control group	Treated group
*EC*_*50*_ *(mg/mL)*	33 ± 2	33 ± 2	22 ± 2	19 ± 3

The antioxidant activity of egg yolks from both the treated and control group appears to be similar, with no statistical difference observed neither at the halfway (p =  0.805), nor at the end of the trial (p =  0.08), indicating that including sea urchin waste into the diet does not affect the production of carotenoids in egg yolks, which are responsible for their antioxidant activity.

Interestingly, there is a notable difference in antioxidant activity between the yolks sampled midway through the experiment (exhibiting a higher EC_50_, indicative of lower antioxidant activity) and those sampled at the end (showing higher antioxidant activity). The ambient temperature, light conditions, and humidity were maintained constant throughout the trial, excluding their influence. Previous studies have noted a decline in egg quality with increasing age of the hens, attributed to reduced antioxidant capacity in the liver and decreased formation of yolk precursors [55–58]. Stress could also be a contributing factor, as stressed hens may exhibit lower performance and reduced feed intake [45]. However, the welfare evaluation conducted in this study indicated that the hens were in a well state of welfare, with few lesions reported.

To gain further insights, additional studies incorporating blood tests and behavioural assessments are warranted.

#### Eggshell thickness.

As previously mentioned, eggshell thickness is a parameter used to evaluate egg quality [59]. In the present study, eggshell thickness was analyzed with GLM considering treatment and time as factor, together with their interaction. This parameter significantly varied during the trial (F_7;368_ =  13.5; p <  0.001), however the eggs from treated group remained thicker than eggs from the control group (F_1;368_ =  41.8; p <  0.001). Furthermore, there was an interaction between time and treatment (F_1;368_ =  4.5; p <  0.001). The marginal means with 95% confidence interval for the treated and control group were respectively 0.38 mm (0.376–0.385) and 0.36 mm (0.354–0.364). The thickness of the treated group was slightly higher than values reported in the literature (0.28–0.36 mm) [52,60]. When eggshell thickness exceeds 0.34 mm, the opacity of the cuticle is more than 27.5%, enhancing eggshell antibacterial efficiency up to 98% [61]. Additionally, Ledvinka et al. [52] reported that thinner eggshells increase permeability, contributing to economic losses and risk of bacterial infection. Thus, an increase in eggshell thickness correlates with improved antibacterial protection [52,61]. The results obtained contrast with findings from Swiatkiewicz et al. [62] and Lee et al. [63], who did not observe differences in eggshell thickness when changing calcium sources. However, magnesium present in sea urchin waste (even in small quantities) could have positively influenced eggshell thickness, as observed in the study by Kim et al. [12].

#### Eggshell breaking strength.

In the study, to investigate whether the biogenic source of calcium affects eggshell breaking strength, quasi-static compression was applied to the equatorial zone, which represents the weakest and most uniform part of the egg, providing a more accurate estimate of the eggs’ resistance [64–66]. The results of t-test for eggshell breaking strength are reported in Table 6.

**Table 6:** Breaking strength.

	*Treated group (mean±SD)*	*Control group (mean±SD)*	*P-value*
*39*^*th*^ *week*	36.4 ± 5.6	37.1 ± 4.9	0.437
*52*^*nd*^ *week*	31.6 ± 4.1	32.2 ± 5.5	0.449

The results of the breaking strength test show no significant difference between the two groups, consistent with findings from Swiatkiewicz et al. [17] and Lee et al. [63], who reported that calcium source does not influence breaking strength. However, Guinotte and Nys [42] found an improvement in egg breaking strength in hens supplemented with ground seashells and oyster shell.

The outcomes obtained at the 39^th^ week are comparable to those reported by Leyendecker et al. [41] and Koreleski and Swiatkiewicz [67]. A decrease in breaking strength was observed with increasing age of the animals, in line with several other studies [67–69].

The structure of the eggshell also influences breaking strength [60]. Rodriguez-Navarro et al. [70] demonstrated a correlation between eggshell strength and crystallographic texture, where about 40% of the variance in shell strength could be attributed to differences in crystal orientation. Ketta and Tůmová [60] reported a positive correlation between eggshell thickness and breaking strength. In contrast, similar to our findings, Ahmadi and Rahimi [71] showed that increased eggshell thickness does not necessarily lead to higher breaking strength, as it is influenced by eggshell construction quality, including ultrastructure and the arrangement of calcite crystals. Therefore, in this study, the ultrastructure was also evaluated (see below). The absence of differences in bone and eggshell breaking strength, alongside a significant increase in eggshell thickness, seem to confirm that calcium derived from sea urchin waste does not negatively affect performance or animal welfare.

#### Ultrastructure.

The ultrastructural organization of the mammillary layer of the eggshell was analyzed using a Scanning Electron Microscope (SEM) to investigate whether supplementing with sea urchin waste had any structural impact on the eggshell and its potential correlation with mechanical performance. As previously mentioned, sea urchin skeletons are primarily composed of calcium carbonate, with a small amount of magnesium carbonate [7]. The presence of magnesium carbonate could positively influence eggshell quality in terms of weight, strength, and thickness, as demonstrated by Belkameh et al. [72] through increased magnesium supplementation in laying hens’ diets. Additionally, differences in the origins of calcium carbonate could affect its intestinal assimilation and thus its bioavailability. Saunders-Blades et al. [18] reported better eggshell quality with oyster shell supplementation compared to limestone, while Solomon [73] described various structures within the mammillary layer, including Type A and B mammillary bodies. These are cup- or cone-shaped protrusions with an irregular (“fibrous”) cap located on the inner side of the eggshell, connecting it to the egg membranes. Fusion of the fibrous caps of the mammillary bodies can influence the formation of the palisade layer and alter pore distribution. Park and Sohn [74] noted that low-density mammillary bodies weaken the eggshell’s resistance.

**Fig 2:**
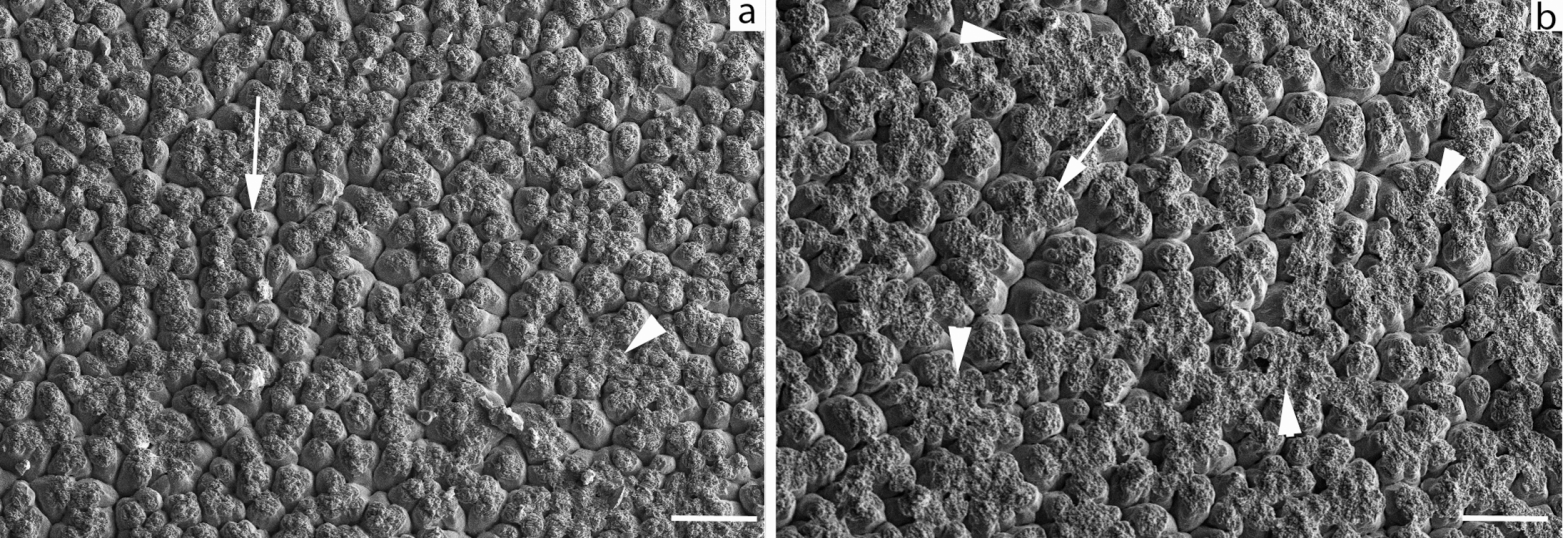
SEM image of the eggshell.

SEM observations of eggshells collected at the 42^nd^ week revealed no significant differences between the control and treated group. However, in some samples of the treated group, there were greater dimensional variations in the mammillary bodies and more frequent zones of fusion between their fibrous caps (Fig 2). Nevertheless, these minor differences did not affect the mechanical resistance of the eggshell, as no differences were found in breaking strength. No differences were observed between the two groups in the eggshells collected at the 51^st^ week (data not shown). Therefore, it is possible to conclude that the supplementation with sea urchin waste did not affect the ultrastructure (mammillary layer) of the eggshells, thus further confirming the outcomes from the mechanical tests.

The figure shows the inner side of the eggshell (42^nd^ week) with its characteristic cup-shaped mammillary bodies (arrows). No remarkable difference or anomalies can be observed between control (A) and treated sample (B), except for a larger amount of areas of fusion of the fibrous cap (arrowhead).

#### Purity and magnesium content.

Considering that sea urchin skeleton contains also magnesium, the X-ray diffraction (XRD) analysis was performed on selected samples to determine the purity and crystalline composition of eggshells. All spectra exhibit a single-phase pattern corresponding to the rhombohedral calcite crystal structure with the diffraction peaks slightly shifted to higher angles indicating shrinkage of the crystal lattice. The reduction of the lattice parameters can be explained by the random incorporation of Mg^2 +^ cations. The molar Mg-content was calculated as described in the Supporting information (S6 File). The absolute values are not significant, but the trends indicated by the data can be felt to be meaningful to evaluate the behaviour of the system.

It was found that the magnesium content varies between 2.5 and 3.0 mol% (2.72 ± 0.15 mean±MAD) for the control samples and between 1.8 and 3.1 mol% (2.46 ± 0.47 mean±MAD) for the treated samples.

The values are spread out over a relative narrow range in both series of samples. The mean values of the two sets are quite similar although the values of the mean absolute deviation (MAD) indicate a greater variability and spread of the data relative to the treated samples.

These results suggest that there is no important effect of diet on Mg content in eggshells. However, the one-factor testing results showed that the difference in behavioural response might not be statistically significant (p =  0.297).

## Conclusion

Substituting limestone-derived calcium (L-Ca) with sea urchin waste-derived calcium (S-Ca) in the diet of laying hens did not significantly affect egg production, but resulted in a lower percentage of discarded eggs in the treated group. Welfare evaluations showed fewer lesions in the treated hens, on the head, back, tail, comb and footpad, with no significant differences in cloaca, keel bone, and tibia breaking strength. This suggests that in the present study the S-Ca diet did not negatively impact animal welfare nor bone quality.

Regarding egg quality, eggs from the treated group were slightly lighter in overall weight, yolk and albumen fraction, but remained within the same commercial category as the control group. While eggshell weight and breaking strength were similar between groups, the treated hens produced eggs with significantly thicker eggshells. There were no differences in the yolk pigmentation, antioxidant activity, and in the ultrastructural organization of the eggshell’s mammillary layer, as suggested by the absence of differences in the eggshell breaking strength.

These initial findings, summarized in Table 7, indicate that S-Ca can effectively replace L-Ca without adversely affecting production, or egg quality, and may enhance certain welfare aspects. However, further studies are recommended to confirm these results.

**Table 7:** Summary of the results.

Parameter	Result
Feather evaluation	^+^
Footpad lesions	^+^
Keel bone deviation	0
Tibia breaking strength	0
Egg production	^+^/-^a^
Discarded eggs	^+^
Egg weight	-^b^
Yolk colour	0
Antioxidant activity of the yolk	0
Eggshell thickness	^+^
Eggshell breaking strength	0
Ultrastructure	0
X-ray Powder Diffraction	0
Antioxidant activity of the diet	0

Table 7 showing the results for each evaluated parameter. The sign “+” to indicate the positive effects of S-Ca, “-” for the negative results, and “0” when no effect was obtained (p < 0.05).

^a^ Egg production is slightly lower in the treated group, but in accordance with the hybrid standard.

^b^ Egg weight was significant slightly higher in the control group, but the difference of 1 g does not change the commercial category.

## Supporting information

S1 Table Analytical components of sea urchin waste.
(DOCX)

S2 Table Mineral analysis of sea urchin waste.
(DOCX)

S3 File Ca and Mg solubility in sea urchin waste.
(DOCX)

S4 File Carotenoids quantification of the diet.
(DOCX)

S4 Fig Carotenoids quantification of the diet.
Regression line corresponding to different concentrations of standard of lutein and their relative absorbance at 445 nm.
(TIF)

S5 File Antioxidant activity of the diet.
(TIF)

S5 Fig Antioxidant activity of the diet.
%ABTS remaining at different feed concentrations (mg/ml).
(DOCX)

S6 File Analysis of X-ray diffraction patterns. (DOCX)
