# Supplementary material for: Sea urchin waste as valuable alternative source of calcium in laying hens’ diet
Source: PLoS One. 2025 Mar 4;20(3):e0314981. doi: 10.1371/journal.pone.0314981 (PMC11878918; doi:10.1371/journal.pone.0314981)
Supplement: S1 Table — (DOCX) [file pone.0314981.s001.docx]

**S1 Table**

| water (%) | 0,99 |
| --- | --- |
| dry substance d.s. (%) | 99,01 |
| ash (% d.s) | 93,50 |
| protein (% d.s.) | 1,25 |
| lipid (% d.s.) | 0,10 |
| fiber (% d.s.) | n.d. |
