# Supplementary material for: Sea urchin waste as valuable alternative source of calcium in laying hens’ diet
Source: PLoS One. 2025 Mar 4;20(3):e0314981. doi: 10.1371/journal.pone.0314981 (PMC11878918; doi:10.1371/journal.pone.0314981)
Supplement: S2 Table — (DOCX) [file pone.0314981.s002.docx]

**S2 Table**

| Element | mg/Kg d.s. |
| --- | --- |
| Ag | <0,1 |
| Al | 70,58 |
| As | 4,20 |
| B | 43,90 |
| Ba | 4,42 |
| Be | <0,07 |
| Ca | **122162,90** |
| Cd | 0,16 |
| Co | 0,12 |
| Cr | 1,71 |
| Cu | 0,54 |
| Fe | 156,12 |
| Hg | <0,07 |
| K | 1115,65 |
| Li | 4,54 |
| Mg | **18204,11** |
| Mn | 15,07 |
| Mo | 0,23 |
| Na | 9364,17 |
| Ni | 0,47 |
| P | 585,98 |
| Pb | <0,7 |
| S | 3954,30 |
| Sb | <0,7 |
| Se | <0,7 |
| Si | 330,03 |
| Sn | 2,99 |
| Sr | 1657,47 |
| Ti | 14,97 |
| Tl | <0,7 |
| V | 1,12 |
| Zn | 3,14 |
