# Supplementary material for: Sea urchin waste as valuable alternative source of calcium in laying hens’ diet
Source: PLoS One. 2025 Mar 4;20(3):e0314981. doi: 10.1371/journal.pone.0314981 (PMC11878918; doi:10.1371/journal.pone.0314981)
Supplement: S3 File — (DOCX) [file pone.0314981.s003.docx]

**S3. Ca and Mg solubility in sea urchin waste**

The calcium and magnesium carbonate solubility assay was performed in vitro. *In vitro* solubility of carbonates (Ca and Mg) was determined through modification literature protocols [75]. Briefly, a precisely weighted amount (around 1 g) of dried sea urchin powder with 7.0 ml of 6M formic acid. After 1 hour under stirring, the suspension was filtered using a Büchner filter and the residual solid was dried at 70 °C for 10 h, cooled and weighed to determine the percent weight loss. Results show a solubility equal to 78%, in line with literature data. Gilani *et al.* [76] reported that the average *in vitro* solubility of limestone samples ranged from 19 to 99%. The in vivo relevance of these assay results has not been ascertained thus far.

**Reference**

1. David LS, Anwar MN, Abdollahi MR, Bedford MR, Ravindran V. Calcium Nutrition of Broilers: Current Perspectives and Challenges. Animals (Basel). 2023 May 9;13(10):1590. doi: 10.3390/ani13101590
2. Gilani S, Mereu A, Li W, Plumstead PW, Angel R, Wilks G, Dersjant-Li Y. Global survey of limestone used in poultry diets: calcium content, particle size and solubility. Journal of Applied Animal Nutrition. 2022;10(1):19-30. doi:10.3920/JAAN2021.0015
