# Supplementary figures and images for: Sea urchin waste as valuable alternative source of calcium in laying hens’ diet
Source: PLoS One. 2025 Mar 4;20(3):e0314981. doi: 10.1371/journal.pone.0314981 (PMC11878918; doi:10.1371/journal.pone.0314981)

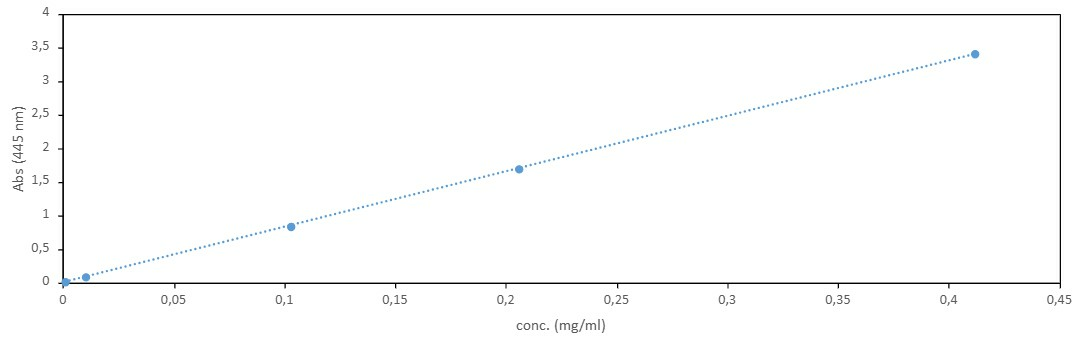

Supplement: S4 File — (DOCX) [file pone.0314981.s004.tif]

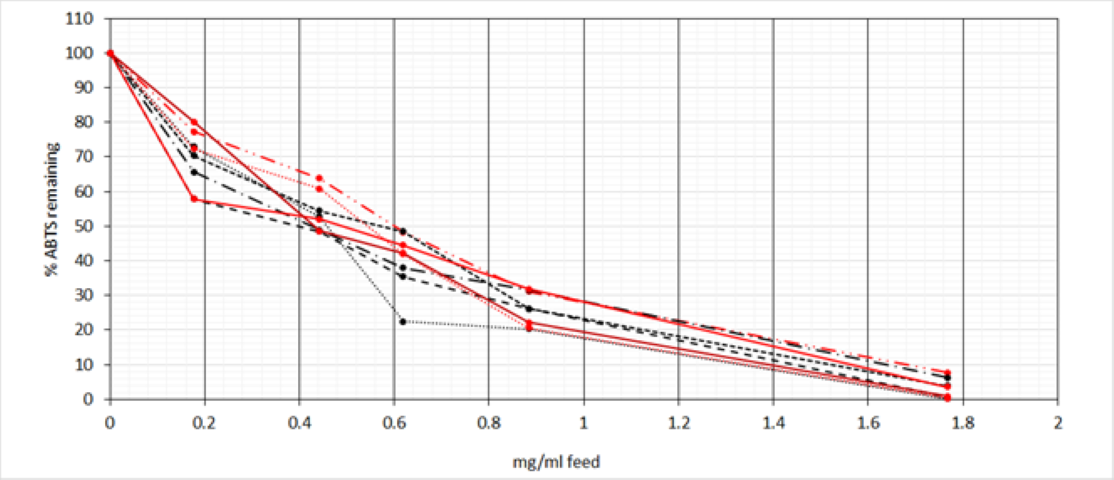

Supplement: S5 File — (TIF) [file pone.0314981.s006.tif]
