# Supplementary material for: Sea urchin waste as valuable alternative source of calcium in laying hens’ diet
Source: PLoS One. 2025 Mar 4;20(3):e0314981. doi: 10.1371/journal.pone.0314981 (PMC11878918; doi:10.1371/journal.pone.0314981)
Supplement: S4 Fig — Regression line corresponding to different concentrations of standard of lutein and their relative absorbance at 445 nm. (TIF) [file pone.0314981.s005.docx]

**S4 File. Carotenoids quantification of the diet**

To better explore the previous aspect about the antioxidant activity of the diets, a specific analysis of the carotenoids contained in each diet was conducted and results were expressed as total carotenoids (lutein equivalents).

Aiming at expressing results in terms of ‘lutein equivalents’ a calibration line was built with lutein commercial standard. Plotting the experimental data ‘[lutein] vs Absorbance at 447 nm’, it was possible to verify that the data exhibit a linear trend, with the linear regression line represented by the following mathematical equation: y = 8.2536x – 0.0007. S1 Fig displays the corresponding linear regression line together with raw data. The correlation coefficient R^2^, equal to 1, confirms the linearity within the acquired data range.

Figure S4

Results indicate the presence of 0.39±0.03 g of total carotenoids (lutein equivalents) per kg of feed in the control diet and 0.42±0.02 g of lutein per kg of feed in the treated diet, again confirming the absence of any statistical difference (p = 0.14) in the total quantity of carotenoids in the two types of diets.

Results from the antioxidant activity and from the carotenoids quantification are justified by the presence in the diets of a relevant amount of additives containing antioxidant compounds such as further carotenoids, that could hide any added value provided by the presence of sea urchins’ secondary metabolites. Among the other ingredients present in both diets, corn is the cereal with the highest concentration of carotenoids, which can vary depending on the hybrid used. The presence of different carotenoids was revealed such as lutein: 6.4-16.0 μg/g, zeaxanthin: 8.3-18.6 μg/g, β-cryptoxanthin: 0.9-3.1 μg/g, β-carotene: 0.6-1.5 μg/g [77]. Also, soft whole wheat represents a source of carotenoids. Examining eight varieties, Moore et al. [78] found the presence of β-carotene: 0.10-0.21 μg/g, zeaxanthin: 0.20-0.39 μg/g and lutein: 0.82-1.14 μg/g.

**References**

1. Meléndez-Martínez AJ, Mandić AI, Bantis F, Böhm V, Borge GIA, Brnčić M, Bysted A, Cano MP, Dias MG, Elgersma A, Fikselová M, García-Alonso J, Giuffrida D, Gonçalves VSS, Hornero-Méndez D, Kljak K, Lavelli V, Manganaris GA, Mapelli-Brahm P, Marounek M, Olmedilla-Alonso B, Periago-Castón MJ, Pintea A, Sheehan JJ, Tumbas Šaponjac V, Valšíková-Frey M, Meulebroek LV, O'Brien N. A comprehensive review on carotenoids in foods and feeds: status quo, applications, patents, and research needs. Crit Rev Food Sci Nutr. 2022;62(8):1999-2049. doi:10.1080/10408398.2020.1867959
2. Moore J, Hao Z, Zhou K, Luther M, Costa J, Yu L. Carotenoid, tocopherol, phenolic acid, and antioxidant properties of Maryland-grown soft wheat. J Agric Food Chem. 2005;53(17):6649-6657. doi:10.1021/jf050481b
