# Supplementary material for: Sea urchin waste as valuable alternative source of calcium in laying hens’ diet
Source: PLoS One. 2025 Mar 4;20(3):e0314981. doi: 10.1371/journal.pone.0314981 (PMC11878918; doi:10.1371/journal.pone.0314981)
Supplement: S5 Fig — %ABTS remaining at different feed concentrations (mg/ml). (DOCX) [file pone.0314981.s007.docx]

**S5 File. Antioxidant activity of the diet**

The assessment of the antioxidant activity of the two diets was conducted to verify whether the feed integrated with sea urchins’ waste could confer a higher antioxidant activity to the feed. This hypothesis is based on what was indicated by Marzorati et al. [7], finding the presence of carotenoids such as echinenone (11±2 mg/kg in tests and spines), astaxanthin 15±1 mg/kg in tests and spines and β-carotene (2.0±0.5 mg/kg in tests and spines), as residuals after gonads removal, characterized by a certain antioxidant power. The same authors also found the presence of polyhydroxynaphthoquinones (PHNQ), specifically Spinochrome B and Spinochrome A, both characterized by high antioxidant power. The ABTS^●+^ quenching results are reported in Fig S2. To compare the data in terms of antioxidant activity, the EC_50_ (concentration of the feed able to induce a 50% quenching of the radical ABTS) for each sample was determined and means and standard deviations for each set (treated and control) were calculated. The EC_50_ for the control diet was found equal to 0.47±0.09, while the EC_50_ for the treated diet was found equal to 0.51±0.08. Results do not show any significant difference (p = 0.505) in the antioxidant activity between the two types of feeds. Therefore, it can be concluded that the two feeds do not present significant differences in terms of antioxidant activity.

Figure S5

Red lines correspond to replicated for the treated diet, while black lines correspond to the replicates for the control diet.

For each investigated sample, it is possible to observe the expected decrease in % of ABTS ^●+^ remaining after 2 hours of reaction at increasing concentrations of feed (mg/ml), indicating a certain activity towards the radical scavenging phenomenon. Higher concentrations of feed correspond to a greater presence of antioxidants that quench the ABTS radical, thus reducing its presence after two hours of reaction.
