# Supplementary material for: Sea urchin waste as valuable alternative source of calcium in laying hens’ diet
Source: PLoS One. 2025 Mar 4;20(3):e0314981. doi: 10.1371/journal.pone.0314981 (PMC11878918; doi:10.1371/journal.pone.0314981)
Supplement: S6 File — (DOCX) [file pone.0314981.s008.docx]

**S6 File. Analysis of X-ray diffraction patterns**

All the spectra showing a shift of the diffraction peaks to higher angles indicating a shrinkage of the crystal lattice were analysed to quantify the magnesium content. The reduction of the lattice parameters can be in fact explained by the random incorporation of Mg^2+^ cations which have a smaller ionic radius (0.72 Å in sixfold coordination) compared to Ca^2+^ (1.00 Å in sixfold coordination) [79].

Analysis and refinement of XRD data was done by using *FullProf* suite software. Lattice parameters and unit cell volume were devised from a full-profile fit of the spectra based on the Le-Bail method [80].

The fitting was performed on all sample between 20 and 65° by variation of key profile and crystallographic parameters, that is lattice parameters, FWHM and peak shape parameters (U, V, W, eta0 and x), background coefficients (6 term polynomial), isotropic temperature factor and zero point. All refinements provided good fits, acceptable reliability factor parameters (chi-square values χ2 for the fit are in the interval 5.36 – 9.57) and lattice parameters with low ESDs.

The Mg-content was calculated from the cell dimensions (a, c and V) extracted from the XRD data according to the Bischoff equation [81]:

*V* = 368.1 – 122x + 131x^2^

*a* = 4.9906 – 0.50x + 0.56x^2^

*c* = 17.069 – 2.27x + 2.1x^2^

*c/a* = 3.420 – 0.118x + 0.05x^2^

where X is the mole fraction of MgCO_3_ in the carbonate. The method is based on Vegard’s law [82] which states that the crystal lattice constants vary linearly with the composition of the constituent elements. The approximation is valid for ideal solutions and Mg-contents in the range 2 – 16 mol% assuming no distortions in the unit cell except for those caused by Mg substitution [83]. The equation related to the cell volume was applied because considered the most reliable.

**References**

1. Shannon RD. Revised Effective Ionic Radii and Systematic Studies of Interatomic Distances in Halides and Chalcogenides. Acta Crystallographica Section A. 1976;32:751-767. doi:10.1107/s0567739476001551
2. Le Bail A. Whole powder pattern decomposition methods and applications: A retrospection. Powder Diffr. 2005;20(4):316-326. doi:10.1154/1.2135315
3. Mackenzie F, Bischoff W, Bishop F. Biogenically produced magnesian calcite: inhomogeneities in chemical and physical properties: comparison with synthetic phases. Am Mineral. 1983;68(11):1183-1188
4. Jacob KT, Raj S, Rannesh L. Vegard's law: a fundamental relation or an approximation? Z Metallkd Mater Res Adv Tech. 2007;98(9):776-779. doi:10.3139/146.101545
5. Chave KE. A solid solution between calcite and dolomite. J Geol. 1952;60(2):190-2. doi:10.1086/625949
